# Hermansky-Pudlak syndrome-associated pneumothorax with rapid progression of respiratory failure: a case report

**DOI:** 10.1186/s12890-020-01302-8

**Published:** 2020-10-06

**Authors:** Yukari Kato, Motoyasu Kato, Hiroaki Ihara, Eri Hayakawa, Kohei Shibayama, Keita Miura, Tomoko Yamada, Yoichiro Mitsuishi, Takehito Shukuya, Jun Ito, Takeshi Matsunaga, Tadashi Sato, Kenji Suzuki, Kazuhisa Takahashi

**Affiliations:** 1 Department of Respiratory Medicine, Juntendo University Graduate School of Medicine, 3-1-3 Hongo, Bunkyo-Ku, Tokyo, 113-8431 Japan; 2 Department of Thoracic Surgery, Juntendo University Graduate School of Medicine, 3-1-3, Hongo, Bunkyo-ku, Tokyo, 113-8431 Japan

**Keywords:** Chest drainage, Hermansky-Pudlak syndrome, Pneumothorax, Pulmonary fibrosis, Pirfenidone

## Abstract

**Background:**

Hermansky-Pudlak syndrome (HPS) is an extremely rare disease with pulmonary fibrosis (PF), oculocutaneous albinism, induced platelet dysfunction, and granulomatous colitis. Although patients with HPS-associated PF (HPS-PF) often receive treatment with anti-fibrotic agents, including pirfenidone, many HPS-PF cases are progressive. The development of pneumothorax is known to be rare in HPS-PF. Pneumothorax development is generally important for prognosis in patients with interstitial pneumonia. However, there are few reports regarding the development of pneumothorax in patients with HPS-PF.

**Case presentation:**

A 50-year-old Japanese man with chestnut hair, white skin, and light brown squint eyes visited our hospital for interstitial pneumonia examination. Chest high-resolution computed tomography (HRCT) demonstrated diffuse bilateral reticular opacities along the bronchovascular bundles and traction bronchiectasis predominantly in the upper lung fields. He was definitively diagnosed with HPS because genetic analysis showed that he had a homozygous mutation, c.398 + 5G > A, in the *HPS-1* gene. After diagnosis with HPS-PF, he initiated home oxygen therapy due to gradually progressive hypoxemia.

Three months after the HPS-PF diagnosis, the patient suddenly developed severe chest pain and dyspnea and was admitted to our hospital on emergency. He was diagnosed with pneumothorax by chest radiological findings. He immediately received chest drainage; however, his pneumothorax did not improve. Therefore, he underwent video-assisted surgery by thoracic surgeons. The leak point was not detected, but multiple bullae were found, mainly in the upper lung lobes. Thus, the surgeons did not perform bullectomy and only covered the apical areas. Fifteen days after the surgery, the patient developed high fever and dyspnea with a new diffuse reticular shadow found through HRCT. We first initiated the patient on broad-spectrum antibiotics; however, the symptoms and radiological findings worsened. Therefore, we started treatment with pirfenidone for inhibition of PF progression. The patient re-developed pneumothorax with severe respiratory failure. Although he re-underwent chest drainage, he died of progressive respiratory failure.

**Conclusions:**

We herein report the case of a rare HPS patient who developed pneumothorax with progressive PF. Pneumothorax may cause rapid progressive respiratory failure and may be associated with PF progression in HPS-PF.

## Background

Hermansky-Pudlak syndrome (HPS) [1] is an exceedingly rare disease with autosomal recessive inheritance and is complicated with oculocutaneous albinism, platelet dysfunction induced by secondary inhibition of platelet aggregation, granulomatous colitis, and pulmonary fibrosis (PF) [2, 3]. The incidence of HPS is one in 500,000 to 1 million. HPS-related PF (HPS-PF) is characterized by usual interstitial pneumonia in pathological findings and slow progression of pulmonary fibrosis in clinical course, with poor prognosis [2]. The prevalence age in HPS is younger compared to that in idiopathic pulmonary fibrosis (IPF). Steroid treatment is reported to be non-effective [4, 5]; therefore, anti-fibrotic agents, including nintedanib and pirfenidone, are usually used to treat patients with IPF, even though these drugs have not been approved for HPS-PF. However, these agents are not commonly used for the treatment of HPS-PF. Although a few patients with HPS-PF have been reported to respond to pirfenidone [6, 7], it did not have a good effect.

The development of pneumothorax is rare in patients with HPS-PF. In general, the development of pneumothorax leads to a decrease in patient quality of life and worsens any respiratory symptoms in patients with interstitial pneumonia.

We herein report the development of severe refractory pneumothorax in a patient with HPS-PF and review the previously published case reports associated with the development of pneumothorax in patients with HPS-PF.

## Case presentation

**Fig. 1:**
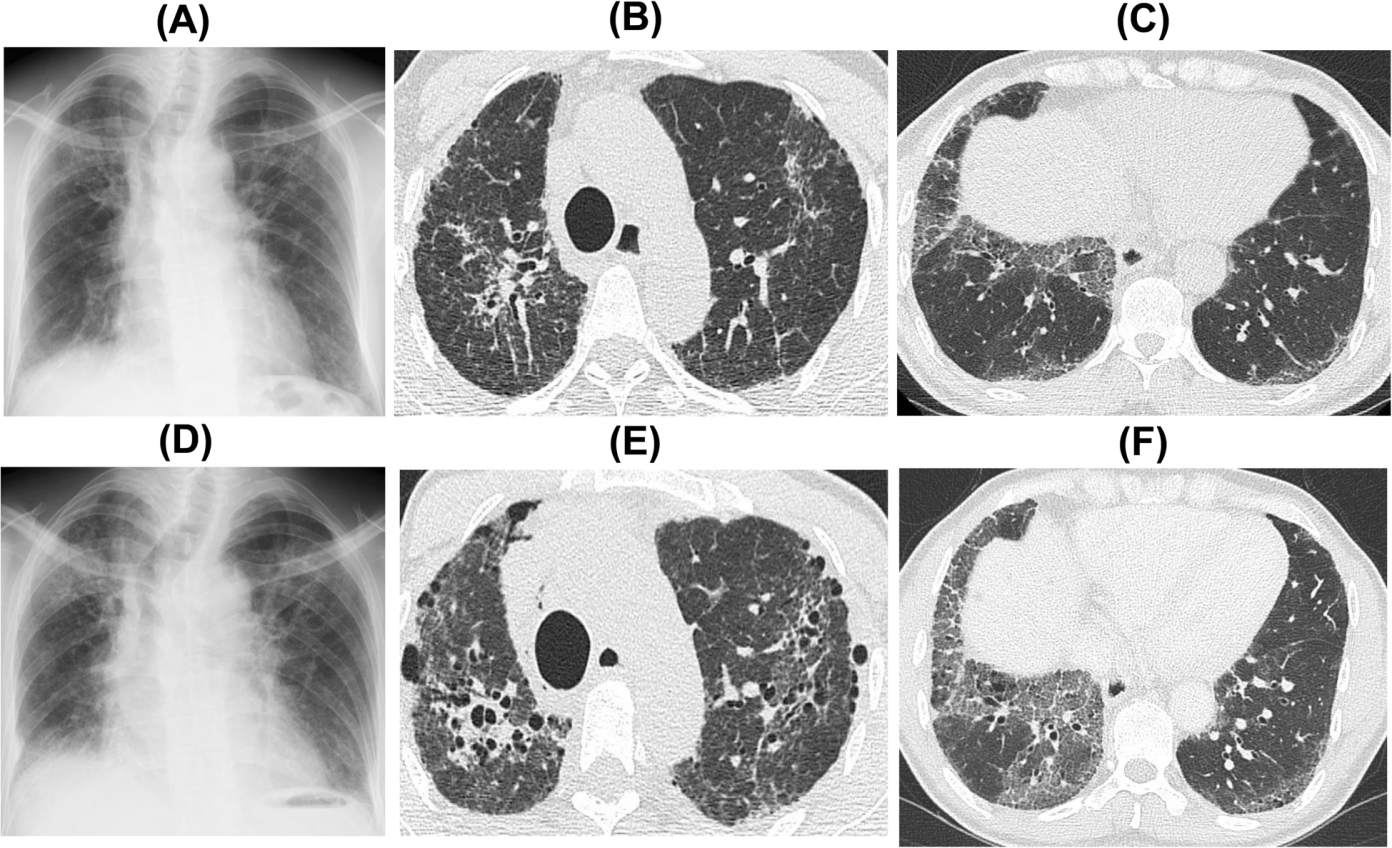
**a** Chest X-ray at the first visit of the patient to our hospital. Chest high-resolution computed tomography (HRCT) findings in the **b** upper and **c** lower lung fields at the first visit to our hospital. **d** Chest X-ray 2 years after the first visit to our hospital. Chest HRCT findings in the **e** upper and **f** lower lung fields 2 years after the first visit to our hospital

A 50-year-old Japanese man, with natural white skin, chestnut hair, and light brown squint eyes, visited our hospital with a suspicion of interstitial pneumonia based on a chest X-ray (Fig. 1a). He had no smoking and history. Further, he had no family history associated with interstitial pneumonia. He worked as an office worker until this admission. High-resolution computed tomography (HRCT) of the chest at the first visit to our hospital showed diffuse bilateral reticular opacities along the bronchovascular bundles and traction bronchiectasis predominantly in the upper lung fields (Fig. 1b and c). These findings on chest X-ray and HRCT gradually progressed in approximately 2 years (Fig. 1d, e, and f). Laboratory data demonstrated that he showed a prolonged bleeding time without decreasing serum platelet number, abnormal coagulation, and elevated levels of Krebs von den Lungen-6 (KL-6) in serum. A pulmonary function test revealed restrictive ventilatory impairment and decreased diffusion capacity for carbon monoxide. He was also diagnosed with oculocutaneous albinism by skin biopsy and ophthalmologic findings. Genetic analysis showed that the patient had a homozygous mutation (c.398 + 5G > A) in the *HPS-1* gene; thus, he was definitively diagnosed with HPS. Further, his interstitial change on HRCT was considered to be associated with HPS-PF. After the diagnosis with HPS-PF, hypoxemia gradually progressed; thus, he started home oxygen therapy (HOT).

**Fig. 2:**
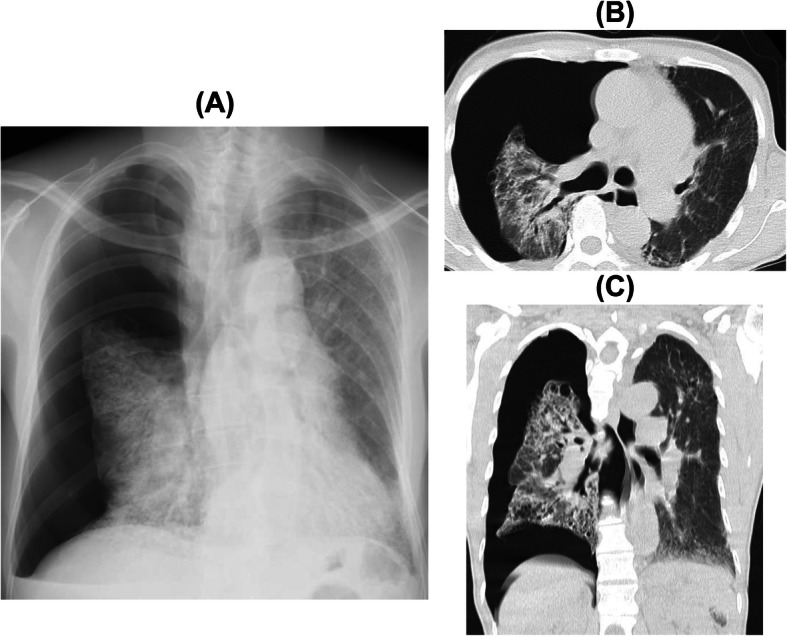
**a** Chest X-ray of the patient upon the development of pneumothorax. **b** Axial and **c** coronal views of chest computed tomography at the development of pneumothorax

**Fig. 3:**
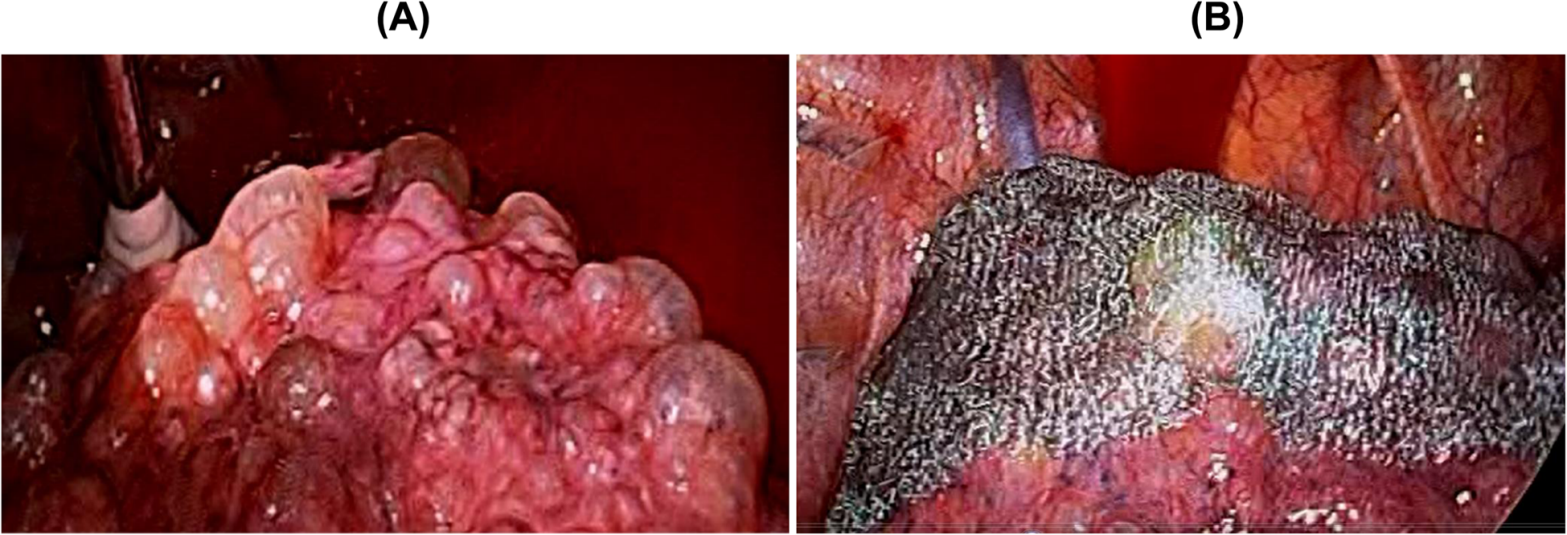
**a** Video-assisted thoracic surgery showed bullae at the lung apex in the right upper lobe of the patient. **b** The right lung apex was covered using absorbable polyglycolic acid felt

Three months after the diagnosis with HPS, the patient was admitted to our hospital on emergency due to a sudden development of chest pain and acutely worsening dyspnea. Chest X-ray showed a collapsed right lung (Fig. 2a), and the patient was diagnosed with secondary pneumothorax caused by the progression of HPS-PF. Findings of the chest HRCT showed a worsening of the reticular shadow with traction bronchiectasis, right lung collapse, and multiple bullae at the lung apex (Fig. 2b and c). We performed chest drainage; however, pneumothorax did not improve. Therefore, the patient underwent video-assisted surgery by thoracic surgeons; the leak point was not detected, but multiple bullae were found, mainly in the upper lung lobes (Fig. 3a). Thus, the surgeons did not perform bullectomy but only covered the apical areas with absorbable polyglycolic acid felt (Fig. 3b). Platelet concentrate was transfused before the operation because of the risk of bleeding associated with the patient’s prolonged bleeding time; the total bleeding was only 20 mL of blood without additional platelet transfusion. After the surgery, the right lung almost fully expanded, and there were no specific operation-induced problems detected within 10 days post-operation. Fifteen days post-operation, the patient developed high fever and complained about dyspnea. HRCT findings demonstrated the appearance of new ground-glass opacities in the right lower lung lobes. We first initiated broad-spectrum antibiotics due to the suspicion of bacterial pneumonia; however, the symptoms and hypoxemia got worse. The reticular opacities also spread gradually on the chest radiograph, along with elevation of the serum KL-6 level. There were no significant findings regarding bacterial infection during this admission. We considered this as the progression of HPS-PF triggered by the development of pneumothorax or by lung surgery. We initiated pirfenidone for the inhibition of HPS-PF progression; however, it was not effective in suppressing HPS-PF progression. On postoperative day 33, pneumothorax recurred in the right lung. Although we re-performed chest drainage, pneumothorax did not improve due to continuous air leakage. Due to severe respiratory failure, we could not re-operate. Forty-two days after pneumothorax diagnosis, the patient died of respiratory failure.

## Discussion and conclusions

Our patient was diagnosed with HPS based on *HPS-1* genetic analysis. Many gene subtypes are reported to be associated with HPS. Patients with *HPS-1*, *HPS-2*, and *HPS-4* often show PF complications [8]. Particularly, patients with *HPS-1* have a high incidence of PF [8] and more progressive fibrosis [9]. Our patient showed *HPS-1* mutation; thus, the findings from our patient are consistent with those from previous cases. Our patient showed complications of oculocutaneous albinism, platelet dysfunction, and a history of granulomatous colitis; hence, our patient had almost all features associated with HPS. This patient developed pneumothorax; however, this manifestation is rare in HPS. During the observation period, PF and the respiratory condition worsened rather rapidly. In general, survival time has been reported to be approximately 30 years in HPS [2]. In contrast, the survival time was 3 months from the diagnosis of HPS in our patient. The prognosis in our case is shorter than that in previous reports because of the rapid progression of PF and the development of pneumothorax.

There have been only two cases reported on the development of pneumothorax in patients with HPS-PF ([10, 11], Table 1). A patient who developed pneumothorax underwent bullectomy and died of respiratory failure 2 years after surgery [10]. Another patient underwent chest drainage and pleurodesis under local anesthesia because of the worsening respiratory condition and died of respiratory failure one year after surgery for pneumothorax [11]. In contrast, our patient first developed pneumothorax three months after diagnosis with HPS-PF and died of respiratory failure 1 month after surgery for pneumothorax. Our patient underwent patch closure to the visceral pleural fistula. The survival time from surgery for pneumothorax was short compared to that in previously reported cases. In all these cases, the patients died of respiratory failure in their fifties. However, lung biopsy could not be performed because of the rapid progression of chronic respiratory failure before surgery; therefore, the prognosis was poorer in our case compared to previous cases. Our patient had already started receiving HOT upon HPS-PF diagnosis. In contrast, the patients in the two published cases did not receive HOT upon the development of pneumothorax. The difference in respiratory conditions and severity of interstitial pneumonia between our case and those previously reported may be associated with the difference in the time from the development of pneumothorax to death.

**Table 1:** The comparison between previous patients and our case

Reference	Age/Sex	Gene type	Time to the development of pneumothorax from diagnosis with interstitial pneumonia	Treatment for pneumothorax	Outcome and cause of death	Time to death from surgery for pneumothorax
Kaseda et al. [10]	59/Male	*HPS-1*	5 years	Bullectomy	Death by respiratory failure	2 years
Kirisi et al. [11]	54/Female	Unknown	2 years	1. Chest drainage 2. Pleurodesis	Death by respiratory failure	1 year
Our case 2020	50/Male	*HPS-1*	2 years	1. Chest drainage 2. Pulmonary fistula closure	Death by respiratory failure	1 month

For the treatment of HPS-PF, due to HPS-PF progression, our patient received pirfenidone. However, lung fibrosis sub-acutely worsened when the patient received the anti-fibrotic agent. We judged that pirfenidone was not effective against PF in our patient. We also considered treatment with other anti-fibrotic agents such as nintedanib. However, the patient had a prolonged bleeding time and pneumothorax; thus, we did not choose this agent due to the possibility of the condition worsening, as well as other adverse effects. Corticosteroids are used for treating any interstitial pneumonia; however, we cannot administer corticosteroids for IPF [12]. HRCT scanning demonstrated a few findings, including ground-glass opacity and consolidation, honeycombing, traction bronchiectasis, and reticular shadow, in our patient. We considered these HRCT findings to be near usual for interstitial pneumonia. Then, our patient developed pneumothorax and underwent lung surgery; thus, we considered the possibility that steroid-induced delayed wound healing might have induced this negative effect. Therefore, we did not select corticosteroids; rather, we chose the anti-fibrotic agent, pirfenidone. Lung transplantation is known as the only life-extending therapy for patients with HPS-PF [13, 14]. In our case, the patient had already been registered for lung transplantation; however, the patient developed pneumothorax and died before his turn for transplantation.

In conclusion, we report here a rare HPS case of pneumothorax development with progressive PF. The development of pneumothorax may cause the rapid progression of respiratory failure and PF. Clinicians should be made aware that pneumothorax development indicates a poor prognosis in patients with HPS-PF.

## Data Availability

Data sharing is not applicable to this article as no datasets were generated or analysed during the current study.

## Abbreviations

IPF: Idiopathic pulmonary fibrosis
HPS: Hermansky-Pudlak syndrome
HPS-PF: Hermansky-Pudlak syndrome-associated pulmonary fibrosis
HOT: Home oxygen therapy
HRCT: High-resolution computed tomography
KL-6: Krebs von den Lungen-6
PF: Pulmonary fibrosis
